# Efficient object location determination and error analysis based on barycentric coordinates

**DOI:** 10.1186/s42492-020-00052-y

**Published:** 2020-07-31

**Authors:** Andrea Bodonyi, Roland Kunkli

**Affiliations:** 1 Faculty of Informatics, University of Debrecen, Debrecen, H-4028 Hungary; 2 Doctoral School of Informatics, University of Debrecen, Debrecen, H-4028 Hungary

**Keywords:** Barycentric coordinates, Conversion, Transformation, Coordinate systems, Location

## Abstract

In this paper, we propose an efficient computational method for converting local coordinates to world coordinates using specially structured coordinate data. The problem in question is the computation of world coordinates of an object throughout a motion, assuming that we only know the changing coordinates of some fixed surrounding reference points in the local coordinate system of the object. The proposed method is based on barycentric coordinates; by taking the aforementioned static positions as the vertices of a polyhedron, we can specify the coordinates of the object in each step with the help of barycentric coordinates. This approach can significantly help us to achieve more accurate results than by using other possible methods. In the paper, we describe the problem and barycentric coordinate-based solution in detail. We then compare the barycentric method with a technique based on transformation matrices, which we also tested for solving our problem. We also present various diagrams that demonstrate the efficiency of our proposed approach in terms of precision and performance.

## Introduction

Conversion between coordinate systems and applying coordinate transformations are common techniques for solving different scientific problems. This general method is efficiently used in various fields, such as computer graphics, image processing [1], geometric processing [2], physics [3], and geoinformatics [4, 5]. In this paper, we propose a method for dealing with a similar problem, where conducting conversions between different coordinate systems is also necessary.

To illustrate our problem, let us assume we have a motion sequence described by each of its steps. What we are interested in is the global position of the moving object in each step. Object determination is a common problem in different scientific fields as well; however, there may be cases where the available information is limited or special in some way [6–9]. In our case, we assume that the given data is special in that only a few reference points (and their changing coordinates), which are located in the surroundings of the moving object and defined in its local coordinate system, are known. To obtain the desired object positioning, we must determine the coordinates of the main object for every step by converting from the object’s local coordinate system to the global one using the information at our disposal. There is also a need for analyzing the provided precision, which is essential in different object locating applications [10, 11].

To this end, we propose a computational method that is capable of obtaining the required motion data using the information outlined above. The essence of the method lies in the use of barycentric coordinates related to the surrounding reference points. Barycentric coordinates are widely used in the field of computer graphics. They have many applications, including interpolation and deformation [12–14], character articulation [15], and mesh parameterization [16, 17]. Their utility has been proved in other fields besides computer graphics, such as sensor networks [8] and robot localization, as a part of the trilateration method [6, 7].

In the next section, we present the problem in detail by describing the data concept. Then, we continue with a brief overview of barycentric coordinates along with a thorough demonstration of our proposed method. After that, we evaluate our method by comparing it with other possible methods, and the same section shows the corresponding error metrics for their precision and performance as well. Lastly, the paper is concluded with a summary of our results.

## Methods

### Problem definition

Our main problem is given by the concept of data creation and what kind of information it provides us. As we mentioned above, it contains a motion series that is described by the environment of the moving object. This supposition implies the task of locating this object in the global coordinate system based on its environment.

In the data sequence, each step contains information that is described only by itself. At every step of the motion, we only know the changed coordinates of the surrounding reference points with respect to the local coordinate system of the main object. Nevertheless, our goal is to compute the (changing) world coordinates of the main object, assuming that the reference points remain fixed in the global system. We also know that the main object starts its motion at the origin of the global coordinate system. Of course, if we consider its local coordinates, the object plays the role of the origin in every step.

In other words, the motion is defined by some base points in the surroundings of the object. Knowing that these reference points are stationary, we can rely on them to retrieve the position of our observed object.

The structure of the data for a motion step in the sequence is built up as follows: it contains a step identifier and an object array that holds four objects. For each object, the array stores the corresponding relative position in the current step. There is no additional information contained in the data.

### Barycentric approach

#### Barycentric coordinates

Barycentric coordinates have the capability of giving the position of an arbitrary point based on some reference points. We can get this mentioned position by taking an adequately weighted sum of the said references. In the following, we present an example case for this in the plane.

Based on ref. [18], we would like to highlight the main characteristics of barycentric coordinates in the three-dimensional case.

Let us consider an arbitrary given tetrahedron with vertices **a**, **b**, **c**, and **d**. Any point on the edge between **a** and **b** can be obtained as (1 – *k*)**a** + *k***b**, where 0 ≤ *k* ≤ 1. Let us assume that point **r** is considered in the mentioned form.

Then—employed the previously used technique—any point **q** of the segment of **r** and **c** can be determined as (1 – *l*)**r** + *l***c**, where 0 ≤ *l* ≤ 1.

Similarly, any point **p** of the segment of **q** and **d** can be calculated as (1 – *m*)**q** + *m***d**, where 0 ≤ *m* ≤ 1. Now, let us express **p** with *k*, *l*, *m*, and the points of the tetrahedron. From the previously mentioned equations, we get

$$ {\displaystyle \begin{array}{l}\mathbf{p}=\left(1-m\right)\mathbf{q}+m\mathbf{d}\\ {}=\left(1-m\right)\left(\left(1-l\right)\mathbf{r}+l\mathbf{c}\right)+m\mathbf{d}\\ {}=\left(1-m\right)\left(1-l\right)\mathbf{r}+\left(1-m\right)l\mathbf{c}+m\mathbf{d}\\ {}=\left(1-m\right)\left(1-l\right)\mathbf{r}+\left(l- lm\right)\mathbf{c}+m\mathbf{d}\\ {}=\left(1-m-l+ lm\right)\left(\left(1-k\right)\mathbf{a}+k\mathbf{b}\right)+\left(l- lm\right)\mathbf{c}+m\mathbf{d}\\ {}=\left(1-m-l+ lm-k+ km+ kl- kl m\right)\mathbf{a}+\left(k- km- kl+ kl m\right)\mathbf{b}+\left(l- lm\right)\mathbf{c}+m\mathbf{d}\end{array}} $$

Let us introduce the following notations:

$$ {\displaystyle \begin{array}{l}t:= 1-k-l-m+ lm+ km+ kl- kl m,\\ {}u:= k- km- kl+ kl m,\\ {}v:= l- lm,\\ {}w:= m.\end{array}} $$

**Fig. 1:**
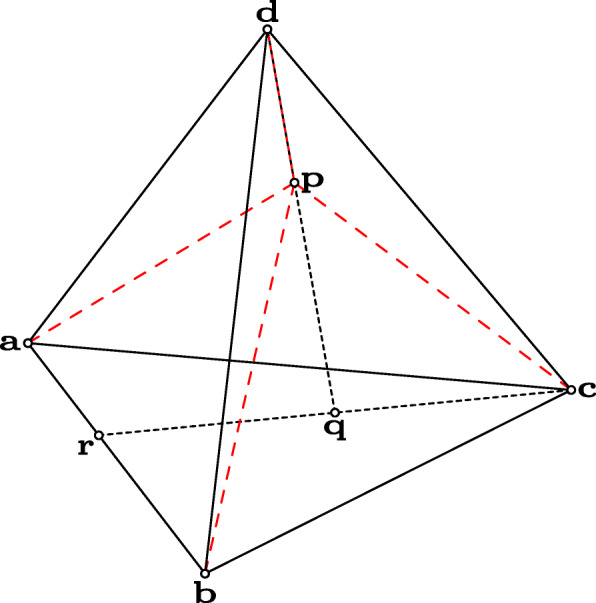
Barycentric coordinates of point p inside the tetrahedron abcd. In the figure, **r** = (1 – 0.4)**a** + 0.4**b**, **q** = (1 – 0.5)**r** + 0.5**c**, and **p** = (1 – 0.6**q**) + 0.6**d**. Using the computation method proposed below to obtain *t, u, v,* and *w* barycentric coordinates of **p**, we get that **p** = 0.12**a** + 0.08**b** + 0.2**c** + 0.6**d**

Using the notations introduced above, **p** can be written as **p** = *t***a** + *u***b** + *v***c** + *w***d**, and it is easy to see that *t* + *u* + *v* + *w* = 1. The *t*, *u*, *v*, and *w* numbers are called the barycentric coordinates of point **p**, and by using them, the point can be calculated as a weighted sum of **a**, **b**, **c**, and **d**. As an example, see Fig. 1. With the proposed calculation based on the intervals between 0 and 1, we can describe only the inner part of the tetrahedron, but this idea can be generalized to any positions in the three-dimensional space.

#### Determining global coordinates using barycentric coordinates

As we pointed out earlier, our computation is based on the use of barycentric coordinates. To use the barycentric approach as a solution to our problem, it is essential to be able to calculate the barycentric coordinates of our moving object with respect to the four fixed reference positions-which surround our moving one-in its local coordinate system.

First, we introduce some notations. Let us assume that *x*_*i,k*_, *y*_*i,k*_, and *z*_*i,k*_ (*i* ∈ {1, 2, 3, 4}) are the *x*, *y*, and *z* coordinates, respectively, of the *i*-th surrounding reference point in the *k*-th step. Furthermore, *w*_*i,k*_ (*i* ∈ {1, 2, 3, 4}) will denote the barycentric coordinates of the main object also in the *k*-th step regarding this reference system. We also know that in this part of our computation, everything is described in the local coordinate system of the main object. Thus, the main object is always located at the origin and has (0, 0, 0) coordinates.

Based on the method suggested on page 46 in ref. [19] for the two-dimensional case, we can reach the desired barycentric coordinates as follows.

Considering that ${\sum}_{i=1}^4{w}_{i,k}=1$, *w*_4*,k*_ can be expressed using the other weights as

1 $$ {w}_{4,k}=1-\sum \limits_{i=1}^3{w}_{i,k} $$

Using this—and also the fact that the observed object’s local coordinates are (0, 0, 0) in every step—we can get the following system of equations for the *k*-th step:

2 $$ {\displaystyle \begin{array}{c}\sum \limits_{i=1}^3{w}_{i,k}\cdot {x}_{i,k}+\left(1-\sum \limits_{i=1}^3{w}_{i,k}\right)\cdot {x}_{4,k}=0\\ {}\sum \limits_{i=1}^3{w}_{i,k}\cdot {y}_{i,k}+\left(1-\sum \limits_{i=1}^3{w}_{i,k}\right)\cdot {y}_{4,k}=0\\ {}\sum \limits_{i=1}^3{w}_{i,k}\cdot {z}_{i,k}+\left(1-\sum \limits_{i=1}^3{w}_{i,k}\right)\cdot {z}_{4,k}=0\end{array}} $$

The equation system (2) trivially leads us to the following:

3 $$ {\displaystyle \begin{array}{c}\sum \limits_{i=1}^3{w}_{i,k}\cdot \left({x}_{i,k}-{x}_{4,k}\right)=-{x}_{4,k}\\ {}\sum \limits_{i=1}^3{w}_{i,k}\cdot \left({y}_{i,k}-{y}_{4,k}\right)=-{y}_{4,k}\\ {}\sum \limits_{i=1}^3{w}_{i,k}\cdot \left({z}_{i,k}-{z}_{4,k}\right)=-{z}_{4,k}\end{array}} $$

which can also be written in matrix form as

4 $$ {A}_k\cdot {\mathbf{w}}_k={\mathbf{b}}_k $$

where

$$ {A}_k:= \left(\begin{array}{ccc}{x}_{1,k}-{x}_{4,k}& {x}_{2,k}-{x}_{4,k}& {x}_{3,k}-{x}_{4,k}\\ {}{y}_{1,k}-{y}_{4,k}& {y}_{2,k}-{y}_{4,k}& {y}_{3,k}-{y}_{4,k}\\ {}{z}_{1,k}-{z}_{4,k}& {z}_{2,k}-{z}_{4,k}& {z}_{3,k}-{z}_{4,k}\end{array}\right) $$

$$ {w}_k:= \left(\begin{array}{c}{w}_{1,k}\\ {}{w}_{2,k}\\ {}{w}_{3,k}\end{array}\right),{b}_k:= -\left(\begin{array}{c}{x}_{4,k}\\ {}{y}_{4,k}\\ {}{z}_{4,k}\end{array}\right) $$

From the matrix form above, we can compute the *w*_*i, k*_ weights as

5 $$ {\mathbf{w}}_k={A}_k^{-1}\cdot {\mathbf{b}}_k $$

Based on Eq. (1), we can easily calculate *w*_4,*k*_ with vector operations as

6 $$ {w}_{4,k}=1-\left\langle {\boldsymbol{w}}_k,\mathbf{1}\right\rangle $$

where 〈,〉 denotes the dot product, and **1** is the (1 1 1)^*T*^ vector.

So far, we have only found the barycentric coordinates of **0** in the local coordinate system of the actual step. Now, we apply these barycentric coordinates to obtain the position of the moving object for the current step in the global coordinate system.

To get the global coordinates of our object, we need to form a static reference basis in the global coordinate system. We form this basis by the position vectors of the four references from the very first step. Considering them as the four vertices of a tetrahedron, they can be used with the barycentric coordinates derived in the actual step to compute the world coordinates of the searched position.

We assumed that the moving object starts its path at the origin of the global coordinate system. Therefore, at the start of the motion, the coordinates of the four reference points are the same as in the local one. Using this fact, we can consider the calculated barycentric coordinates from every step and use them as weights for the starting reference positions.

Let us collect the barycentric coordinates from the *k*-th step into vector ${\hat{\mathbf{w}}}_k$, i.e.,

$$ {\hat{\mathbf{w}}}_k:= {\left({w}_{1,k}\kern0.75em {w}_{2,k}\kern0.75em {w}_{3,k}\kern0.75em {w}_{4,k}\right)}^T $$

Let us assume that *G*_*i*_ is the matrix containing the local coordinates of the reference positions in the following form:

7 $$ {G}_i:= \left(\begin{array}{cccc}{x}_{1,i}& {x}_{2,i}& {x}_{3,i}& {x}_{4,i}\\ {}{y}_{1,i}& {y}_{2,i}& {y}_{3,i}& {y}_{4,i}\\ {}{z}_{1,i}& {z}_{2,i}& {z}_{3,i}& {z}_{4,i}\end{array}\right) $$

If we use this notation for the start of the movement, the global coordinates of the reference positions can be collected in *G*_1_, and they are the same as their local coordinates at the beginning.

Now we have all necessary data to obtain the desired solution—the global coordinates in the actual step—as the weighted sum of the reference positions. If these coordinates are denoted by *x*_*k*_, *y*_*k*_, and *z*_*k*_, they can be calculated as follows:

8 $$ \left(\begin{array}{c}{x}_k\\ {}{y}_k\\ {}{z}_k\end{array}\right)={G}_1\cdot {\hat{\mathbf{w}}}_k $$

### Transformation matrix-based alternative

When trying to determine a suitable way to retrieve the required coordinates, we ran some tests with a few other methods. However, the huge number of motion steps resulted in computational errors with increasing tendencies throughout the entire simulation in most cases.

The most promising basic method could be using matrix transformations to get the proper coordinates. In each step, we considered the four reference positions from two consecutive steps from our motion data (the previous and current ones) and calculated the matrix that transforms the first set of positions into the second one. We obtained the final transformation by multiplying the previous matrix (computed using the same algorithm) with the current one and used the result to retrieve the desired object location for the current step.

Using the notation introduced in Eq. (7), we would like to find a matrix for which the following is true:

9 $$ {T}_{k+1}\cdot {G}_k={G}_{k+1} $$

i.e., *T*_*k* + 1_ is the transformation matrix that transforms the reference positions from the *k*-th step to the *k* + 1-th. Therefore, we can compute the transformation matrices in the following way:

10 $$ {T}_{k+1}={G}_{k+1}\cdot {G}_k^{-1} $$

Based on this formulation and given our assumption that the object is initially located at the origin, we can obtain the position of the observed object in the *i*-th motion step by accumulating all of the transformation matrices until that step. Formally written, the desired position of the object is

11 $$ \left(\prod \limits_{k=1}^i{T}_k^{-1}\right)\cdot {\left(0\kern0.5em 0\kern0.5em 0\kern0.5em 1\right)}^T $$

Equation (11) can be rearranged to have only one inverse matrix calculation in the following way:

12 $$ {\left(\prod \limits_{k=0}^{i-1}{T}_{i-k}\right)}^{-1}\cdot {\left(0\kern0.5em 0\kern0.5em 0\kern0.5em 1\right)}^T $$

## Results

To have ground-truth reference information to compare our results with and to have a measure for obtaining the accuracy of the two methods, we generated some test data sequences describing different movement types of the object. These synthetic sequences differ in the movement alignment of the object for analyzing the impact of each motion type on the approximation errors.

**Fig. 2:**
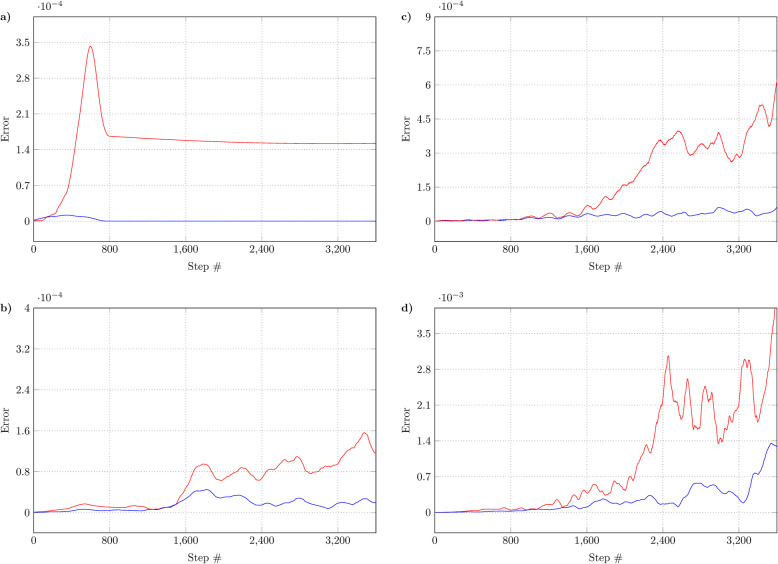
Precision measurements. The blue line marks the precision of the approach based on barycentric coordinates, while the red line marks the precision of the method using matrix transformations. Each measurement differs in the type of movement: **a** seldom-changing constant direction, **b** spiral movement, **c** constant direction with randomly generated rotations, and **d** randomly generated rotations and translation vectors

Here, we present four unique test cases with various translation and rotation configurations. In the first case, the object moves in a seldom-changing direction with no additional rotation. Following this, the moving object follows a spiral path introduced along the *x*-axis. For the third case, we further varied the first test case having a nearly constant moving direction. We introduced per-step random rotations along the *x* and *y* axes. Finally, we kept the random rotations from the previous test case but randomized the translation vectors in each step too. For each test case, we collected the absolute errors of the matrix method and barycentric approach as the distance between the results provided by each method and the ground-truth information. We used these numbers as performance indicators of the individual test approaches. We summarize the resulting error metrics in Fig. 2.

### Precision

As demonstrated by our results, the error produced by the matrix transformation method is several orders of magnitude larger than that of our proposed algorithm. Because barycentric coordinates yield an error of approximately 10^− 6^, it is difficult to visualize the error on the same scale as the matrix transformation error. For this reason, the error of the barycentric method may appear as zero in some places, but the magnitude difference between the two methods is clearly noticeable.

In the case of periodic movement, where the object had no rotation and seldom changed its moving direction, we experienced a nearly constant error amount with a magnitude of 10^− 4^ (Fig. 2a). In the case when the object was moving on a spiral path with and without random rotations (Fig. 2b and c), the error was first relatively unified at a magnitude of 10^− 5^ with little growth, eventually reaching a magnitude of 10^− 4^. This means that the matrix method produced acceptable results. The next test case, however, shows a different view. In this case, the object moves in randomly generated directions with per-step random rotations (Fig. 2d). Such a complicated scenario resulted in larger-scale errors having a firmly rising tendency, showing a growing precision difference between the barycentric method and matrix transformations.

### Performance

We also conducted performance measurements for the two methods. The results show a clear gap between the barycentric approach and the method using matrix transformations. We had three runs for the runtime measurement, containing 10000, 100000, and 200000 steps. We measured the times for the process of calculating the position of the main object for all motion steps.

The results of different cases show a rising tendency for the difference between the runtime of the two methods. With 10000 and 200000 steps, the program run gives us a nearly six millisecond and 240 millisecond difference, respectively. In our case, this means more than 200% runtime growth (see the exact values in Table 1). We ran the performance tests on a dual-core Intel Core i5-3230M 2.60GHz processor.

**Table 1:** Performance measurements of the barycentric approach and matrix method

Step count	Barycentric approach	Matrix method
10000	14.5 ms	20.7 ms
100000	54.9 ms	85.9 ms
200000	101.4 ms	343.5 ms

## Discussion and future work

In this paper, we proposed a method to obtain the actual position of an object using some reference points from its surroundings. Our computation builds upon barycentric coordinates and uses the available points as references to obtain the movement of the observed object in the global coordinate system.

The strength of the barycentric method lies in the fact that information from earlier motion steps is seldom used, leading to reduced error propagation in the system. On the other hand, as mentioned before, in three dimensions, four reference points are required to retrieve the barycentric coordinates of a point. We had four guaranteed fixed points that we could use as references for the barycentric coordinates, which gave a perfect basis for designing the proposed method.

As the self-localization problem is always dependent on the actual environment, it always requires special solutions that account for the available information or that are measurable with disposable devices. Thomas outlined the problem of robot localization in their work [6], from which a possible use case can be derived. As a robot moves, its sensors may collect coordinate information about the surrounding static objects relative to itself; this results in a data format from which the movement-dependent localization problem can be easily solved using the method proposed in this paper.

Additionally, the suggested barycentric method can also solve the inverse of the problem presented in ref. [20]. If we can find static anchors in the surroundings of a moving pedestrian such that we can determine the positions of the anchors relative to them, one could determine the global position of the pedestrian using our proposed algorithm.

Lastly, the field of scientific visualization is another great use case for the proposed barycentric position determination algorithm. We can mainly imagine the algorithm helping with the visualization and animation of object motion data originating from specialized, domain-specific simulation systems.

Considering the results presented in this paper, we plan to work on the applications of the barycentric method in motion data from simulations of an ongoing biological study. We would also like to examine and resolve special cases, such as possible changes in the current fixed environment. We also plan to evaluate the computations in more detail by applying new special cases and a homogeneous coordinates-based computation approach [21] and making further precision measurements of them.

## Data Availability

The datasets used in this study are available from the corresponding author on reasonable request.
